# Retraction: Hyaluronan Activation of the Nlrp3 Inflammasome Contributes to the Development of Airway Hyperresponsiveness

**DOI:** 10.1289/ehp.1205188RET

**Published:** 2015-07-01

**Authors:** John W. Hollingsworth

**Affiliations:** Division of Pulmonary, Allergy, and Critical Care Medicine, Department of Medicine, Duke University School of Medicine, DUMC 103004, 106 Research Dr., Durham, NC 27710 USA. Telephone: (919) 684-4588. E-mail: john.hollingsworth@duke.edu

Feifei Feng, Zhuowei Li, Erin N. Potts-Kant, Yiming Wu, W. Michael Foster, Kristi L. Williams, and John W. Hollingsworth

Environ Health Perspect: http://dx.doi.org/10.1289/ehp.1205188RET

Online: 1 July 2015

Retraction of: Environ Health Perspect 120:1692–1698; doi:10.1289/ehp.1205188

The authors request retraction of “Hyaluronan Activation of the Nlrp3 Inflammasome Contributes to the Development of Airway Hyperresponsiveness.” Due to concerns about the initial data from the animal physiology laboratory at Duke, the authors re-exported source data from the Flexivent machine and compared these data with raw data originally received from the animal pulmonary physiology laboratory. Results from these initial comparisons suggested potential inconsistencies in the data. Therefore, the authors proceeded to replicate experiments of animal airway physiology presented in Figure 2A. These replicate experiments failed to validate the originally reported role of Nlrp3, ASC, and caspase 1 in airway hyperresponsiveness after exposure to ozone. These observed inconsistencies significantly impact the overall conclusions of the manuscript. Therefore, all of the authors have agreed to retract this paper in an effort to correct the scientific record. The authors sincerely apologize for any inconvenience to the scientific community.

